# Quercitrin-nanocoated titanium surfaces favour gingival cells against oral bacteria

**DOI:** 10.1038/srep22444

**Published:** 2016-03-01

**Authors:** Manuel Gomez-Florit, Miguel A. Pacha-Olivenza, Maria C. Fernández-Calderón, Alba Córdoba, Maria L. González-Martín, Marta Monjo, Joana M. Ramis

**Affiliations:** 1 Group of Cell Therapy and Tissue Engineering, Research Institute on Health Sciences (IUNICS), University of Balearic Islands, Palma de Mallorca, Spain; 2 Instituto de Investigación Sanitaria de Palma, 07010 Palma, Spain; 3 Networking Research Center on Bioengineering, Biomaterials and Nanomedicine (CIBER-BBN), Badajoz, Spain; 4 Department of Applied Physics, Faculty of Science, University of Extremadura, Av. Elvas s/n, 06071 Badajoz, Spain; 5 Department of Biomedical Sciences, University of Extremadura, Badajoz, Spain

## Abstract

Many dental implants fail due to the infection and inflammation that walk hand in hand with poor healing and soft tissue integration. Titanium surfaces were nanocoated with quercitrin, a natural flavonoid, with the aim to improve soft tissue integration and increase dental implants success. *Streptococcus mutans* attachment and biofilm formation was analysed. Then, the anti-inflammatory properties and the potential of quercitrin-nanocoated surfaces to boost soft tissue regeneration were tested using human gingival fibroblasts. An inflammatory situation was mimicked using interleulin-1-beta. We found that quercitrin-nanocoated surfaces decreased initial bacterial adhesion while increasing human gingival fibroblasts attachment. Furthermore, quercitrin-nanocoated Ti increased collagen mRNA levels and decreased matrix metalloproteinase-1/tissue inhibitor of metalloproteinanse-1 mRNA ratio, which is related to a reduced metalloproteinase-mediated collagen degradation, while also decreasing the pro-inflammatory prostaglandin E_2_ release under basal and inflammatory conditions. These results suggest that quercitrin-nanocoated surfaces could enhance the soft tissue integration and increase dental implants success.

Success of a dental implant depends on osseo- and soft tissue-integration. Many dental implants fail due to the infection and inflammation that walk hand in hand with poor healing and soft tissue integration^1^,^2^. Soft tissue integration to dental implants establishes a biological seal between the oral environment and the implant which prevents microbial invasion, gingival recession and inflammatory peri-implantitis^2^,^3^,^4^. Therefore, the development of antibacterial, anti-inflammatory and tissue-regenerative surface modifications constitutes a major challenge to guarantee implant success.

Research efforts to develop a new generation of dental implants are focused in bioactive surface coatings intended to provoke a defined cellular reaction^5^. A number of modified surfaces with integrated antibiotics, growth factors, antiresorptive drugs, synthetic peptides or complex formulations of artificial extracellular matrix (ECM) components have been developed. However, biocompatibility of most anti-bacterial surfaces is still uncertain while the lack of stability of growth factors after implantation is the main reason for the absence of such a product from the current implant market^6^,^7^.

An interesting option for surface modification is the use of natural multi-target molecules. Natural molecules, which promise higher therapeutic efficacy and safety^8^, represent an alternative to pharmaceuticals and animal-derived compounds due to low immunogenicity and toxicity^9^. Flavonoids are natural phenolic compounds derived from plants with antioxidant^10^, anti-inflammatory^11^ and antimicrobial capacity^12^,^13^, among other functions^9^. In previous studies, we screened among different flavonoids and selected quercitrin due to its potential in soft and hard periodontal tissues regeneration and its anti-inflammatory activity^14^. Furthermore, we successfully grafted quercitrin to titanium (Ti) surfaces, producing a quercitrin-nanocoating, and observed that the resulting surfaces have promising biological activities *in vitro*^15^. In a posterior work, to increase the nanocoating stability and reproducibility, quercitrin grafting conditions were optimized (Fig. 1). The optimized quercitrin-nanocoated Ti surfaces enhance human mesenchymal stem cells initial adhesion and promote osteoblast differentiation^16^. One step closer for the exploitation of quercitrin-nanocoated Ti-surfaces for their use in dental implant abutments would be the evaluation of their antibacterial, anti-inflammatory and soft tissue-regenerative properties.

In the present study, we aimed at evaluating the effects of quercitrin-nanocoated Ti-surfaces in bacterial adhesion and biofilm formation using *Streptococcus mutans*. Furthermore, human gingival fibroblasts were used to evaluate the effects of quercitrin-nanocoated Ti-surfaces in cell adhesion, anti-inflammatory properties and soft tissue regeneration in normal and in inflammatory microenvironments.

## Results

### Bacterial adhesion and biofilm formation

**Figure 2:**
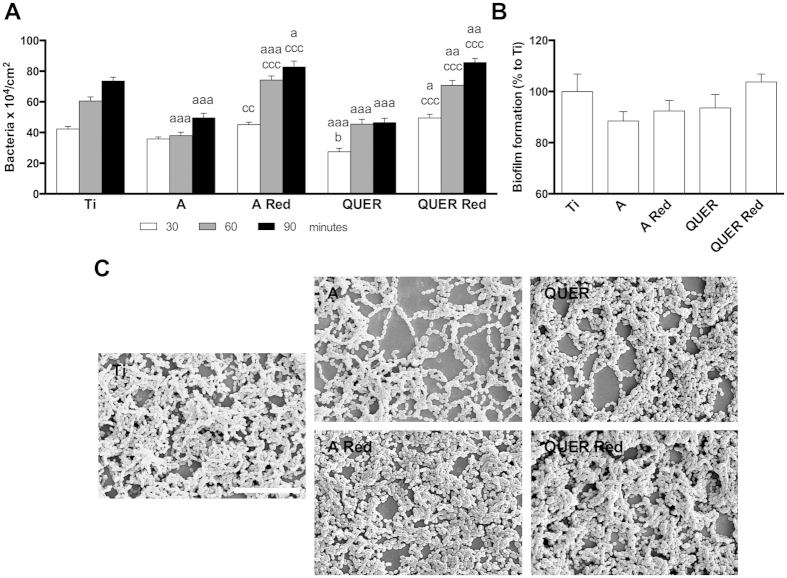
Bacterial adhesion and biofilm formation. (**A**) Dinamic bacterial adhesion: quantitative analysis of the live bacteria adhered to the control and modified surfaces after 30, 60 and 90 min. (**B**) Biofilm formation: quantitative analysis of the percentage of biofilm formation on the different surfaces after 24 hours. (**C**) Scanning electron microscope images of the biofilm formed on the different surfaces (scale bar = 100 μm). One, two and three symbols represent a significant difference between two groups with P ≤ 0.05, P < 0.01 and P < 0.001, respectively: (a) versus Ti control at each time point; (b) versus A control at each time point; (c) effect of reduction within each group at each time point.

QUER surfaces significantly decreased *S. mutans* adhesion after 30 min compared to Ti and A controls. After 60 and 90 min A and QUER surfaces decreased bacterial adhesion compared to Ti control. Unexpectedly, A Red and QUER Red surfaces increased bacterial adhesion at all time points compared to Ti control and to non-reduced surfaces (Fig. 2). Moreover, in all surfaces studied and times, the bacterial viability was not compromised.

We did not find statistical differences in biofilm formation between the different groups. However, A and QUER groups decreased biofilm formation by 12 and 6% respectively, compared to Ti control (Fig. 2). Higher biofilm formation was observed for A Red and QUER Red surfaces compared to the non-reduced surfaces. Furthermore, SEM images confirmed these trends (Fig. 2).

Due to the increased bacterial adhesion of A Red and QUER Red surfaces, we decided to select A and QUER surfaces for further analysis.

### Cell adhesion

**Figure 3:**
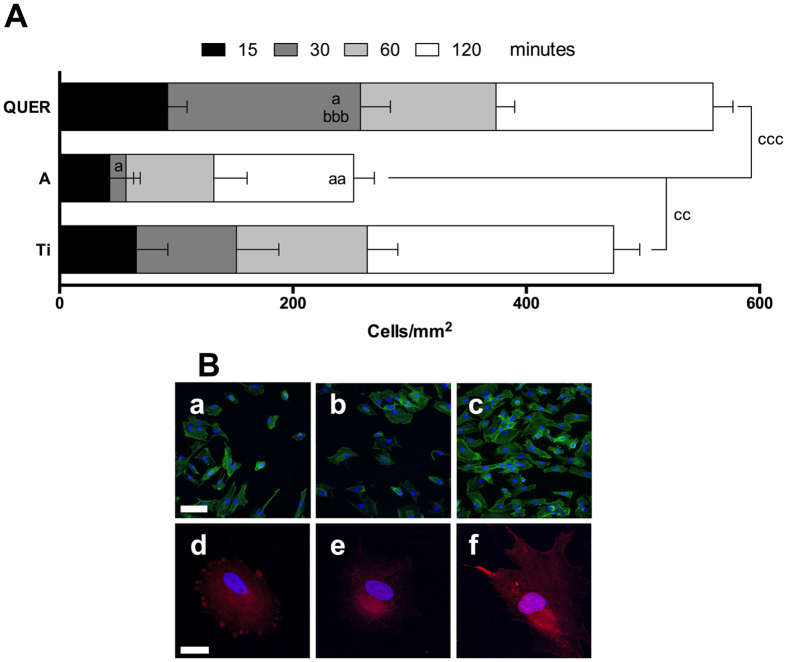
Adhesion of hGF to the different surfaces. (**A**) Quantitative analysis of the cells adhered to the different surfaces after 15, 30, 60 and 120 minutes. (**B**) Cells were allowed to adhere to the surfaces for 30 min. Then, cells were stained with phalloidin-FITC (a–c; green) or with anti-vinculin antibodies (d–f; red) and nuclei were stained with DAPI (blue). Ti (a,d); A (b,e); QUER (c,f) surfaces (scale bars: upper = 100 μm; bottom = 25 μm). One, two and three symbols represent a significant difference between two groups with P ≤ 0.05, P < 0.01 and P < 0.001, respectively: (a) versus Ti for each time point; (b) versus A for each time point; (c) the total number of cells on the different surfaces was compared.

We found that QUER surfaces increased hGF adhesion compared to Ti and A controls after 30 min although we did not observe this clear effect at longer adhesion times, i.e. 60 and 120 min (Fig. 3). When comparing the total number of cells adhered to the surfaces, A surfaces decreased cell number by almost the half while QUER surfaces increased it almost 20% compared to Ti. Furthermore, QUER surfaces increased cell adhesion compared to A control surfaces.

Confocal images reveal that after 30 min, hGFs on QUER surfaces showed the typical spindle-shaped fibroblastic morphology while on Ti and A surfaces were more rounded. Also, hGFs on QUER surfaces showed higher vinculin staining and filopodia than cells on Ti and A surfaces (Fig. 3).

### Response of hGF to the different surfaces

**Figure 4:**
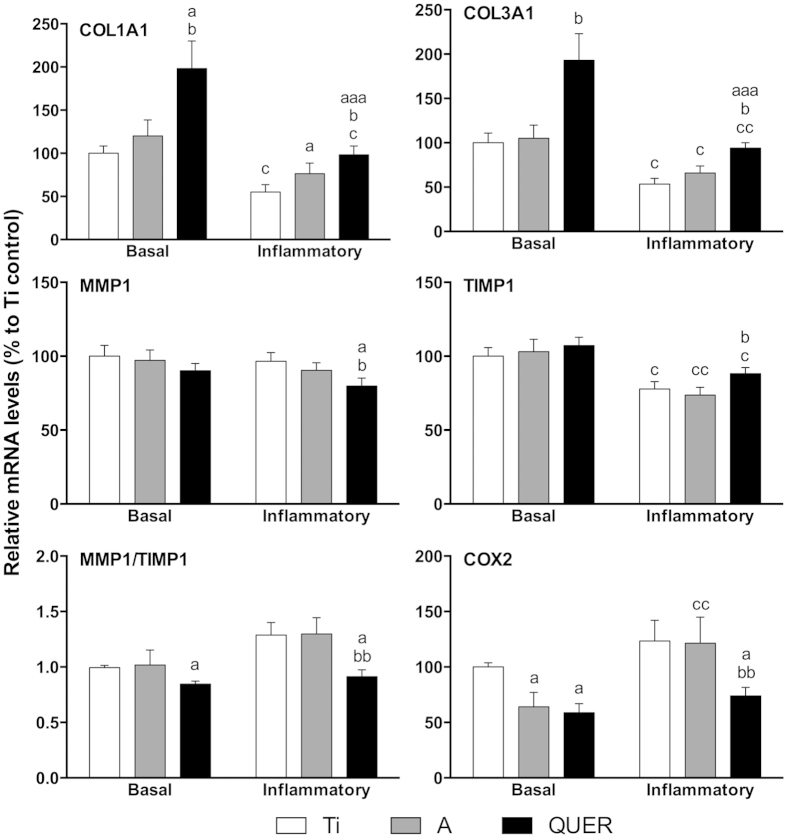
Gene expression of hGF on the different surfaces. Gene expression analysis was performed after 14 days of cell culture. Cells were cultured with IL-1β to mimic inflammation (inflammatory group) or without it (basal group). One, two and three symbols represent a significant difference between two groups with P ≤ 0.05, P < 0.01 and P < 0.001, respectively: (a) versus Ti within each condition; (b) versus A within each condition; (c) effect of IL-1β addition for each surface.

In basal conditions, QUER surfaces increased collagens mRNA levels compared to Ti and A controls and decreased MMP1/TIMP1 mRNA ratio and COX2 mRNA levels compared to Ti. When hGF growing on Ti surfaces were challenged with IL-1β, we observed decreased collagens and TIMP1 mRNA levels while increasing COX2 mRNA. Under this challenging condition, QUER surfaces increased the mRNA levels of collagens while decreasing MMP1/TIMP1 mRNA ratio and COX2 mRNA compared to Ti and A controls (Fig. 4).

**Figure 5:**
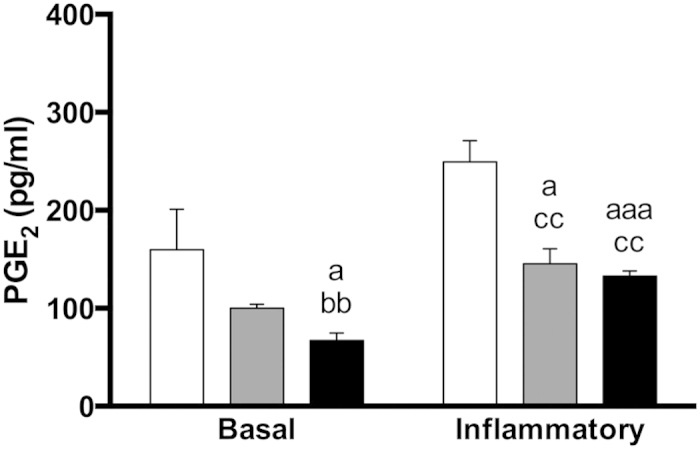
PGE2 release of hGF on the different surfaces. The release of PGE2 to the culture media was evaluated after 14 days of cell culture. Cells were cultured with IL-1β to mimic inflammation (inflammatory group) or without it (basal group). One, two and three symbols represent a significant difference between two groups with P ≤ 0.05, P < 0.01 and P < 0.001, respectively: (a) versus Ti within each condition; (b) versus A within each condition; (c) effect of IL-1β addition for each surface.

After 14 days of cell culture, PGE2 released to culture media was increased on the groups cultured under inflammatory conditions (Fig. 5). Furthermore, A and QUER surfaces decreased PGE2 production, compared to Ti surfaces.

### Effect of disinfection on the bioactivity of the different surfaces

We hypothesized that the disinfection process with dry heat at 100 °C would not affect the coatings bioactivity since this temperature is lower than the melting temperature of quercitrin (176–179 °C) and lower than the boiling temperatures of APTES and quercitrin (223 °C and 316 °C respectively). To confirm this hypothesis, we compared the response of hGF on disinfected (heated) surfaces to non-heated surfaces (see Supplementary Fig. S1). Disinfection process did not affect coatings bioactivity since we only found that COX2 mRNA on Ti disinfected surfaces was decreased compared to Ti non-heated surfaces.

## Discussion

In this study we present a surface nanocoated with quercitrin, a natural flavonoid with multi-target actions, designed to improve soft tissue integration and to increase dental implants long-term success. Quercitrin-nanocoated Ti surfaces decreased initial bacterial adhesion while increasing human gingival fibroblasts attachment. Furthermore, quercitrin-nanocoated surfaces increased collagen mRNA levels and decreased MMP1/TIMP1 mRNA ratio while decreasing COX2 mRNA and PGE2 release under basal and inflammatory conditions. These results suggest that quercitrin-nanocoated surfaces could enhance the soft tissue integration and increase dental implants success.

Peri-implant soft tissue healing follows the four overlapping phases of wound healing^3^. After an implant placement a blood coagulum is formed which provides a provisional ECM. Inflammatory cells migrate, phagocytise foreign particles and release inflammatory mediators to either finalise the inflammation or to amplify it. Fibroblasts then invade the fibrin network and produce collagen fibres to form a connective tissue in contact with the implant surface. The final phase involves collagen remodelling which can produce either a scarred (repaired) tissue or a structural and functional regenerated tissue, depending on the microenvironmental signals. During this process, bacterial accumulation (dental plaque formation), may trigger the inflammatory process. However, it depends on the soft tissue integration to resist the deletereous effects of plaque accumulation, thus, avoiding peri-implantitis to guarantee long-term efficacy of dental implants.

Peri-implant tissue is colonized by the same flora as the periodontium^17^. Among oral micro flora, *S. mutans* is one of the initial colonizers during dental plaque formation, which prepare a favourable environment for the late colonizers, and it is a recognised biofilm forming specie^18^. We found that quercitrin-nanocoated surfaces inhibited initial *S. mutans* attachment, in line with the results obtained on glass and hydroxyapatite surfaces using *S. mutants* suspensions in tea extract solutions (containing flavonoids, tannins and indolic compounds)^19^. Furthermore, biofilm formation was slightly inhibited on quercitrin-nanocoated surfaces. In previous studies, quercitrin had a bacteriostatic effect on *Staphylococcus epidermidis*^14^ while Hasan *et al.*^20^ found that quercitrin decreased *S. mutans* biofilm formation. However, in both studies quercitrin concentrations were much higher than the amount of biomolecule grafted to our surfaces, which was 0.2 to 0.8 nmol of quercitrin per Ti coin on QUER surfaces^16^. It is interesting to highlight that aminosilanized surfaces (A group) also decreased bacterial adhesion, in agreement with previous reports^21^. It has been shown that bacterial adhesion depends on the surface’s terminal functionality and that, in fact, decreases on amino (NH_2_)-terminated surfaces^22^. Further studies on anti-bacterial properties of surfaces should confirm the present results.

Our surfaces reduced with sodium cyanoborohydride (Red groups) showed an increased bacterial adhesion and biofilm formation. In a previous work, the amount of quercitrin grafted on QUER Red surfaces was around 0.1 nmol per Ti coin, less than that grafted to the non-reduced QUER surfaces^16^. The hydrolytic stability of siloxanes at pH 7.5 has been reported to be poor^23^. Therefore, the grafting conditions used to produce QUER Red and A Red samples, at pH 7.5, may favour the hydrolisis of APTES grafted to Ti; on one hand, decreasing the availability of amino-terminal groups on A Red surfaces, and, on the other hand, decreasing the amount of quercitrin that can be grafted on QUER Red surfaces, all in all, increasing bacterial adhesion and biofilm formation. For these reasons, we decided to select QUER surfaces for further analysis.

After installation of a dental implant, gingival fibroblasts are the preferred cells to repopulate the wound and to form a collagen-rich connective tissue^24^. We found that quercitrin-nanocoated surfaces increased initial hGF attachment. This fact together with the decreased bacterial attachment on quercitrin-nanocoated surfaces, increase hGF possibilites to win the “race for the surface” against oral bacteria^25^ since it depends on both an increased biomaterial surface area cell coverage and a decreased number of bacteria present on the surface^26^,^27^. Compared with often-reported monofunctional surface coatings on which bacterial adhesion and biofilm formation is discouraged or they promote host tissue integration^7^,^28^, quercitrin-nanocoated surfaces decrease bacterial adhesion while increasing cell attachment. Furthermore, quercitrin-nanocoated surfaces avoid toxicity concerns of some anti-bacterial coatings. Therefore, the surfaces presented herein may form the required connective tissue around the implant to establish the biological seal that could prevent further bacterial colonization and ultimately preventing the implant loss by peri-implantitis.

Inflammation is necessary for the effective defence against pathogens and to set in motion tissue repair following injury^29^. However, excessive inflammation has been shown to delay healing and to result in increased scarring, compromising tissue regeneration^30^. Thus, a controlled inflammatory process after an implant placement is critical for their success. Here, we found that quercitrin-nanocoated surfaces effectively inhibited COX2 expression and decreased its functional product PGE2 under basal and inflammatory conditions. Furthermore, the antioxidant properties of quercitrin^14^ could also contribute to the inflammation resolution since oxidative stress is considered one of the major causes of inflammation and inhibition of tissue regeneration^31^.

Collagen synthesis and remodelling is a requisite for complete wound healing. Here we found that quercitrin-nanocoated surfaces increased COL1A1 and COL3A1 mRNA levels in basal and inflammatory conditions. Moreover, in inflammatory conditions MMPs are upregulated while TIMPs, their inhibitors, are downregulated, all together boosting collagen degradation^32^. Quercitrin-nanocoated surfaces decreased MMP1 and increased TIMP1 mRNA levels in inflammatory conditions, thus decreasing inflammation-driven ECM-destruction. Noteworthy, quercitrin decreased the expression of pro-fibrotic markers in a wound healing *in vitro* model^14^ and higher proportions of type III collagen are related to more-regenerative than more-scarring responses^33^. Thus, data suggest that quercitrin-nanocoated surfaces may induce connective tissue formation around a dental implant even in the inflammatory conditions found after a dental implant installation. Furthermore, in previous studies of our group using mesenchymal stem cells, quercitrin-nanocoated surfaces increased cell adhesion and osteoblastic differentiation^16^. Therefore, quercitrin-nanocoated surfaces could also increase the osseointegration process of dental implants. Further clinical studies should confirm the potential of quercitrin-nanocoated surfaces to enhance soft and hard tissue integration of dental implants.

In concusion, nanocoating of titanium surfaces with quercitrin decreased initial bacterial adhesion and increased human gingival fibroblasts attachment. Furthermore, quercitrin-nanocoated Ti increased collagen mRNA levels and decreased MMP1/TIMP1 mRNA ratio while also decreasing COX2 mRNA and PGE2 release under basal and inflammatory conditions. These results are in agreement with previous studies where we found that quercitrin had positive effects on cells from soft and hard tissue when added to the culture media^34^. From a clinical perspective, quercitrin-nanocoated surfaces could increase dental implants efficacy by enhancing peri-implant soft tissue regeneration and decreasing peri-implantitis. Furthermore, this study constitutes an example that quercitrin, and flavonoids in general, could be used in combination with other biomaterials, i.e. hydrogels or scaffolds required in severe cases of periodontal and peri-implant diseases.

## Methods

### Materials

Machined Ti disks, c.p. grade IV, 6.2 mm diameter and 2 mm height were purchased from Implantmedia (Lloseta, Spain). (3-Aminopropyl)triethoxysilane (APTES), quercitrin and sodium cyanoborohydride (NaCNBH_3_) were purchased from Sigma–Aldrich (St. Louis, MO, USA).

### Surface nanocoatings

Surfaces were prepared as previously described (Table 1)^15^,^16^. Briefly, Ti disks were passivated by immersion in 30% (v/v) HNO_3_ for 30 min followed by immersion in H_2_O for 24 h (Ti group). Immediately after Ti passivation, aminosilanization was performed with 2% (v/v) APTES solution in dry toluene for 24 h under a dry nitrogen atmosphere. Then, disks were gently rinsed with dry toluene, acetone, and ethanol, and finally dried under a nitrogen flow. Aminosilanised Ti disks (A group) were then immersed either in quercitrin (1 mM) at pH 5.0 (QUER group), in NaCNBH_3_ (100 μM) at pH 7.5 (A Red group) or in quercitrin (1 mM) and NaCNBH_3_ (100 μM) at pH 7.5 (QUER Red group) aqueous solutions. Samples were stirred for 1 h (150 rpm), gently rinsed with water, and dried under a nitrogen flow. NaCNBH_3_ was used to reduce the imine bond, resulting from the reaction between the terminal amino group of APTES and the ketone group of quercitrin, to a single -C–N- bond to decrease bond reactivity and increase coating reproductibility^16^. This reduction is known to be selective for imine bonds at mildly basic pH. For bacterial experiments, surfaces were polished and cleaned^35^ prior to surface modification. Some samples were disinfected with dry heat (100 °C, 30 min) previous to bacteria/cell seeding.

### Bacterial experiments

The bacterial strain used in this study was *Streptococcus mutans* ATCC 25175 (*S. mutans*). The strain was maintained in brain heart infusion (BHI; Sigma–Aldrich) agar plates and cultured in BHI broth for 24 h at 37 °C and 5% CO_2_.

#### Bacterial adhesion

Bacterial suspension was homogenized through a 3 min ultrasonic bath (Ultrasons; JP Selecta, Barcelona, Spain) and harvested by centrifugation, washed two times with artificial saliva^36^ preconditioned at 37 °C, and resuspended at a concentration of 3 × 10^8^ bacteria/ml in artificial saliva. The flow system was a modified Robbins device where disks were fixed to removable ports that allow contact between the surfaces under study and a flow of suspended bacteria in laminar conditions. Before each experiment, the whole system was filled with artificial saliva and preconditioned at 37 °C. Afterward, the bacterial suspension was allowed to flow through the system at a flow rate of 2 ml/min, corresponding to a shear rate of 0.97 s^−1^ at the wall of the flow chamber (unpublished results). This shear rate is such that the specific interactions that could occur between the cells and the different substrata dominate over the drag force of the fluid flow. The bacterial suspension was perfused through the system with recirculation for 30, 60 and 90 min. Samples were carefully removed from the flow chamber and bacteria were stained with the kit Live/Dead Baclight L-7012 (Invitrogen, Camarillo, CA, USA) according to the manufacturer. Then, 5–6 pictures at randomly chosen locations from each sample were taken under fluorescence and the number of bacteria per unit area was counted. The experiments were run in triplicate from separately grown bacterial suspensions.

#### Biofilm formation

Biofilm formation was quantified as previously described^37^. Briefly, bacteria were resuspended at a concentration of 3 × 10^8^ bacteria/ml in BHI. Ti-modified surfaces were disinfected and placed in 96-well plates in sterile conditions. Then, bacterial suspension (200 μl) was cultured on the surfaces at 37 °C and gentle shaking (20 rpm). After 90 min, bacterial suspension was carefully removed and fresh BHI was added. Samples were cultured at 37 °C and gentle shaking (20 rpm) for 24 h. Biofilm formation was quantified using BacTiter-Glo (Promega, Fitchburg, WI, USA) according to the manufacturer’s instructions. Culture media was removed and BacTiter-Glo (200 μl) was added to the wells and incubated (10 min). Then, the content of each well was transferred to wells 96-well white polystyrene flat-bottomed microtiter plates. The light emission (luciferin-luciferase reaction) was measured in a microplate reader. Each assay was performed in duplicate and repeated two times from separately grown bacterial suspension in order to confirm reproducibility.

#### Scanning electron microscopy

To visualise biofilm formation on the surfaces, bacteria were fixed with glutaraldehyde in PBS (4%, 2 h) and washed with distilled water. At 30 min intervals, the samples were dehydrated by the addition of 50%, 70%, 90% and 100% ethanol solutions. Samples were left at room temperature to evaporate the remaining ethanol before sputter gold coating. A scanning electron microscope (SEM; Hitachi S-3400 N, Krefeld, Germany) using secondary electrons, low vacuum conditions and 15 kV of voltage was used to acquire images.

### Cell culture

Primary human gingival fibroblasts (hGF) were purchased from Provitro GmbH, Berlin, Germany). Three different donors were used (range 19–47 years; male:female ratio 2:1). Provitro assures that cells were obtained ethically and legally and that all donors provided written informed consent. Cells were routinely cultured at 37 °C and 5% CO_2_, and maintained in fibroblast growth medium (Provitro GmbH) supplemented with foetal calf serum (10%; Provitro GmbH), amphotericin (50 ng/ml) and gentamicin (50 μg/ml). Experiments were performed with hGF between passages 7 and 8 after isolation and media was supplemented with ascorbic acid (100 μM; Sigma-Aldrich).

The different coins were placed in 96-well plates in sterile conditions. Three replicates for each donor were seeded at a density of 7.0 × 10^3^ cells on each coin (n = 9). For cell adhesion experiments, four replicates from one randomly selected donor were used (n = 4).

In order to create inflammatory conditions, interleukin-1 beta (1 ng/ml) (IL-1β; R&D systems, Abingdon, UK) was added 1 d after cell seeding and kept until day 3, according to previous studies^34^.

### Cell adhesion and vinculin immunostaining

Once seeded, hGFs were allowed to adhere for 15, 30, 60 and 120 minutes to the different surfaces. Then, unbounded cells were removed by washing twice with PBS. Cells were fixed with paraformaldehyde (4%, 15 min) and permeabilised with Triton X-100 (0.25%, 10 min). For nucleus counting, cells were stained with Phalloidin-FITC (5 μg/ml, 30 min). For vinculin immunostaining, cells were blocked with bovine serum albumin (5%, 1 h; Sigma-Aldrich) followed by incubation with anti-vinculin recombinant rabbit monoclonal antibody (4 μg/ml, 3 h, Invitrogen) and then labelled with Alexa Fluor® 488 goat anti-rabbit IgG secondary antibody (5 μg/ml, 30 min, Thermo Scientific, Rockford, IL, USA). Samples were then mounted with DAPI-Fluoroshield (Sigma-Aldrich) and visualized under a fluorescence microscope and a confocal microscope. For nucleus counting, two pictures from the same positions of each coin were taken. Cells were counted using ImageJ software (National Institutes of Health, Bethesda, MD, USA).

### RNA isolation and real-time RT-PCR analysis

After, 14 days of cell culture, total RNA was isolated using Tripure (Roche Diagnostics, Mannheim, Germany), according to the manufacturer’s protocol. Total RNA was quantified at 260 nm using a nanodrop spectrophotometer (NanoDrop Technologies, Wilmington, DE, USA). The same amount of RNA (0.2 μg) was reverse transcribed to cDNA (42 °C, 60 min) according to the protocol of the supplier (High Capacity RNA-to-cDNA kit, Applied Biosystems, Foster City, CA, USA). Aliquots of each cDNA were frozen (−20 °C) until the PCR reactions were carried out.

Real-time PCR was performed for two reference genes, glyceraldehyde-3-phosphate dehydrogenase (GAPDH) and beta-actin (ACTBL2), and target genes (Table 2). Real-time PCR was performed in a thermocycler (Lightcycler 480, Roche Diagnostics) using SYBR green detection. Each reaction contained 7 μl of master mix (Lightcycler 480 SYBR Green I Master, Roche Diagnostics), the sense and the antisense specific primers (0.5 μM) and cDNA sample (3 μl) in a final volume of 10 μl. The amplification program consisted of a pre-incubation step for denaturation of the template cDNA (5 min 95 °C), followed by 45 cycles consisting of a denaturation step (10 s 95 °C), an annealing step (10 s 60 °C) and an extension step (10 s 72 °C). After each cycle, fluorescence was measured at 72 °C. A negative control without cDNA template was run in each assay.

All samples were normalized by the geometric mean of the expression levels of ACTBL2 and GAPDH and fold changes were related to the control groups using the following equation: ratio = E_target_^ΔCp target (mean control – sample)^/ E_reference_^ΔCp reference (mean control – sample)^ adapted from^38^, where Cp is the is the crossing point of the reaction amplification curve and E is the eficiency from the given slopes using serial dilutions, as determined by the software (Lightcycler 480 software, Roche Diagnostics). Stability of reference genes was calculated using a statistical tool (BestKeeper software, Technical University of Munich, Weihenstephan, Germany)^39^.

### Prostaglandin E_2_ quantification

A commercially available enzyme-linked immunosorbent assay kit was run to quantify prostaglandin E_2_ (PGE2; Thermo Scientific) from cell culture media according to supplier instructions.

### Statistical analysis

All data are presented as mean values ± standard error of the mean (SEM). The Kolmogorov-Smirnov test was done to assume parametric or non-parametric distributions. Differences between groups were assessed by paired t-test or Wilcoxon test, depending on data distribution. Two-way ANOVA test using Fisher’s LSD comparisons was used for adhesion experiments. SPSS software (version 17.0, Chicago, IL, USA) and GraphPad Prism (version 6, La Jolla, CA, USA) were used. Results were considered statistically significant at P ≤ 0.05. One, two and three symbols represent a significant difference between two groups with P ≤ 0.05, P < 0.01 and P < 0.001, respectively.

## Additional Information

**How to cite this article**: Gomez-Florit, M. *et al.* Quercitrin-nanocoated titanium surfaces favour gingival cells against oral bacteria. *Sci. Rep.* **6**, 22444; doi: 10.1038/srep22444 (2016).

## Supplementary Material

Supplementary Information

## Figures and Tables

**Figure 1:**
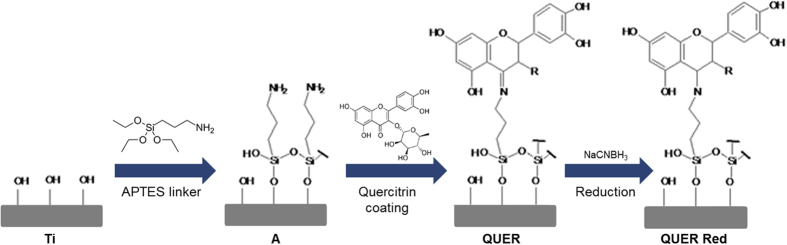
Schema of surface nanocoatings. Ti, titanium passivated surfaces; A, aminosilanised surfaces; QUER, quercitrin-nanocoated surfaces; QUER Red, reduced quercitrin-nanocoated surfaces.

**Table 1:** Groups included in the experiment.

Group	Treatment
Ti	Titanium passivated
A	Ti + APTES
A Red	Ti + APTES + NaCNBH_3_
QUER	Ti + APTES + QUER
QUER Red	Ti + APTES + QUER + NaCNBH_3_

**Table 2:** Sense (S) and antisense (A) primers used in the real-time PCR of reference and target genes.

Gene	Primer sequence (5′–3′)	Product size (bp)
Beta-actin (ACTBL2)	S: CTGGAACGGTGAAGGTGACA	136
	A: AAGGGACTTCCTGTAACAATGCA	
Collagen I α1 (COL1A1)	S: AGAGCATGACCGATGGATTC	122
	A: TTCTTGAGGTTGCCAGTC	
Collagen III α1 (COL3A1)	S: GGCCTACTGGGCCTGGTGGT	190
	A: CCACGTTCACCAGGGGCACC	
Cyclooxygenase-2 (COX2)	S: ATGGGGTGATGAGCAGTTGT	221
	A: GAAAGGTGTCAGGCAGAAGG	
Glyceraldehyde 3-phosphate dehydrogenase (GAPDH)	S: TGCACCACCAACTGCTTAGC	87
	A: GGCATGGACTGTGGTCATGAG	
Matrix metalloproteinase-1 (MMP1)	S: TGTCAGGGGAGATCATCGGGACA	177
	A: TGGCCGAGTTCATGAGCTGCA	
Metalloproteinase inhibitor-1 (TIMP1)	S: TTCCGACCTCGTCATCAGGG	144
	A: TAGACGAACCGGATGTCAGC	
