# Supplementary material for: Quercitrin-nanocoated titanium surfaces favour gingival cells against oral bacteria
Source: Sci Rep. 2016 Mar 1;6:22444. doi: 10.1038/srep22444 (PMC4772538; doi:10.1038/srep22444)
Supplement: Supplementary Information [file srep22444-s1.doc]

**Supplementary Information**

**Quercitrin-nanocoated titanium surfaces favour gingival cells against oral bacteria**

Manuel Gomez-Florit, Miguel A. Pacha-Olivenza, Maria C. Fernández-Calderón, Alba Córdoba, Maria L. González-Martín, Marta Monjo and Joana M. Ramis


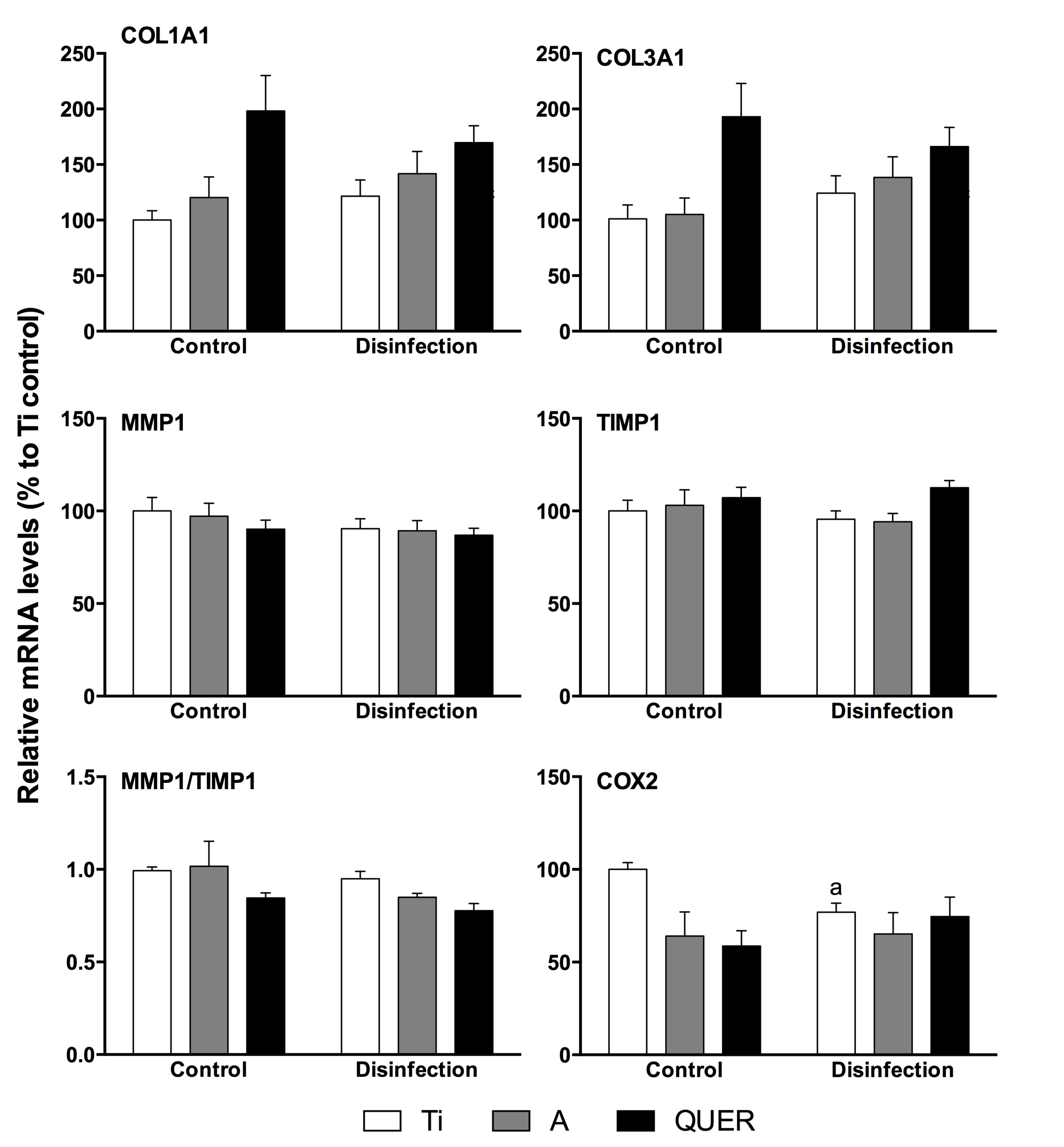


**Supplementary Figure 1. Effect of disinfection on the bioactivity of the different surfaces:** Analysis of gene expression after 14 days of hGF culture on the different surfaces. Cells were cultured on control (non-heated) and disinfected (heated) surfaces. One, two and three symbols represent a significant difference between two groups with P ≤ 0.05, P < 0.01 and P < 0.001, respectively: (a) versus Ti within each condition; (b) versus A within each condition; (c) effect of IL-1β addition for each surface.
